# Rejuvenation of Mature *Ilex paraguariensis* Plants Through Serial Rooted Cuttings: Exploring the Roles of miRNAs in Reversing Adult Phase, Promoting Root Formation, and Determining Root Structure

**DOI:** 10.3390/plants14111668

**Published:** 2025-05-30

**Authors:** María J. Duarte, Raúl M. Acevedo, Nicolás L. Ortiz, Mayra Y. Álvarez, Pedro A. Sansberro

**Affiliations:** Laboratorio de Biotecnología Aplicada y Genómica Funcional, Instituto de Botánica del Nordeste (IBONE-CONICET), Facultad de Ciencias Agrarias, Universidad Nacional del Nordeste, Sgto. Cabral 2131, Corrientes W3402BKG, Argentina; maria.duarte@agr.unne.edu.ar (M.J.D.); rm.acevedo@agr.unne.edu.ar (R.M.A.); nicolas.ortiz8875@gmail.com (N.L.O.); mayra.alvarez@agr.unne.edu.ar (M.Y.Á.)

**Keywords:** *Ilex paraguariensis*, rejuvenation, miRNAs, adventitious rooting

## Abstract

In plants, the transition from the juvenile to adult stage involves physiological and anatomical changes initiated and partially controlled by evolutionarily conserved microRNAs. This process is of particular significance for the successful propagation of woody plant species that have transitioned to vegetative maturity and are recalcitrant to propagation. Conserved miRNAs differentially expressed between rejuvenated and mature *Ilex paraguariensis* plants were identified using high-throughput sequencing of small RNA libraries. The expression of miR156/miR157/miR528 was high in the leaves of juvenile plants and gradually decreased as the plant transitioned from juvenile to adult stages. In contrast, miR172 was predominantly expressed in adult plants. This variation confirmed that adults transitioned back to a juvenile phase after serial-rooted cuttings, allowing the plants to regain juvenile characteristics. Rejuvenation promotes the formation of adventitious roots and improves root structure, which supports the overall growth of the plant and results in greater vigour. The results will offer insights for further investigation into the molecular mechanisms regulating vegetative phase change in *I. paraguariensis* and other recalcitrant woody plant species. This knowledge could facilitate the earlier identification of rejuvenated material by analysing a wider range of genotypes and maturation stages, enhancing the efficiency of *Ilex paraguariensis* mass propagation.

## 1. Introduction

*Ilex paraguariensis* St. Hil. (Aquifoliaceae), known as yerba mate, is a subtropical tree extensively cultivated in monoculture in S Brazil, NE Argentina, and E Paraguay [1]. The dried leaves and thin branches are used to make commercial products that are then utilised to prepare a tea-like infusion with many pharmacological properties beneficial for human health [2,3]. Additionally, its leaves are rich in polyphenols, suggesting potential applications in the food, beauty, and animal breeding industries [4]. Yerba mate is widely consumed in various forms, and its commercialisation is expanding globally. In order to address the continually growing demand, it is imperative to develop new cultivars that enhance tolerance to abiotic and biotic stress, desirable chemical composition, and other pivotal traits. In this sense, numerous protocols utilising in vitro tissue culture [5,6] or greenhouse conditions [7,8,9,10] have been reported for propagating *I. paraguariensis* from stem cuttings. However, despite considerable efforts, mass propagation of adult plants remains challenging.

In perennial woody species, ageing is the main factor constraining the propagation of genotypes with desirable characteristics. Following seed germination, plants undergo a series of developmental stages from the juvenile phase to adult and, ultimately, reproduction [11]. Throughout this life cycle, plants experience notable alterations in morphology and physiology, including reduced vigour, higher susceptibility to abiotic and biotic stressors, and loss of competence for asexual propagation. During the phase transition, plants shift from vegetative to reproductive growth, converting the vegetative shoot apical meristem into an inflorescence meristem. In annual plants, the changes associated with the phase transition are predominantly unidirectional; in perennial plants, the transition alternates between adult vegetative and reproductive phases [12]. Contrariwise, plants that reach the adult or reproductive stage can regain their juvenile characteristics through rejuvenation by artificial methods, including somatic embryogenesis, successive grafts, serial rooted cuttings, and severe pruning [13].

The specific mechanisms that control gene expression during the transition from the vegetative phase are not fully understood. However, research suggests that different types of small RNAs play a significant role in this complex process [14]. Among them, microRNAs (miRNAs, 20–24 nucleotide length) regulate plant development by controlling target gene expression at the post-transcriptional level. Consequently, they are involved in various developmental processes in plants, including the transition to the adult stage, development of shoot apical meristem, leaf formation, floral organ development, and flowering time [15]. In particular, two evolutionary conserved miRNAs, miR156 and miR172, and their target genes have been identified as key components in the genetic control mechanisms regulating plant phase changes. The microRNA miR156 acts by repressing a group of genes known as *SQUAMOSA-PROMOTER BINDING PROTEIN-LIKE* (*SPL*) [16]. These *SPL* genes are crucial for promoting the transition from juvenile to adult stages and initiating flowering [17]. In contrast, miR172 targets mRNAs that code for proteins containing two APETALA 2 (AP2) DNA-binding domains [18]. These proteins play a significant role in regulating the transition to flowering and the development of flowers. During the juvenile stage, the expression of miR156 is prominent in leaves and gradually diminishes in the adult phase across various annual and perennial species [19]. The elevated levels of miR156 promote adventitious root formation in maize, tomato, and tobacco, suggesting that SPL transcription factors inhibit this process [20]. Moreover, *EgSPL2* and *EgSPL5* are up-regulated in mature *Eucalyptus grandis* cuttings compared to juvenile ones, and the latter shows a higher percentage of adventitious rooting [21]. These findings imply that miR156 and miR156 putative targeted *MxSPL* genes may play vital roles in adventitious root formation during the transition from the juvenile to adult phase.

Recent studies have identified small RNAs that respond to various phytohormones, connecting multiple hormonal pathways and creating new links in plant hormonal crosstalk [22]. Yan and colleagues [23] conducted an extensive review discussing the essential mechanisms of miRNAs, their relationships with transcription factors, and the target gene-mediated hormone interactions in regulating root growth and development. Furthermore, Li et al. [24] reported that the expression levels of the miRNAs and target genes are associated with auxin signal-related (miR160 and miR390), stress response-related (miR395, miR398 and miR408), cell fate modification-, proliferation- and enlargement-related (miR156, miR166, miR171, miR319 and miR396) during adventitious root development in apple. However, while these studies provide some initial insights into the mechanisms of adventitious root initiation by specific miRNAs, further research is needed to understand the variation in the transcriptomic profile of miRNAs during rejuvenation in *Ilex paraguariensis*, a difficult-to-root species. Furthermore, it is crucial to elucidate the impact of miRNA expression modification on developing adventitious roots.

Here, we conducted a study to examine how miRNA expression changes in mature leaves of *Ilex paraguariensis* plants of the same genotype but different maturation stages (“ages”) obtained through serial rooting of stem cuttings. We also investigated the relationship between this variation and the competence of softwood cuttings treated with indole-3-butyric acid (IBA) to form adventitious roots. Lastly, we analysed the effect of rejuvenation on plant growth.

## 2. Results

### 2.1. The Effects of Rejuvenation on Adventitious Root Development and Plant Growth

**Figure 1:**
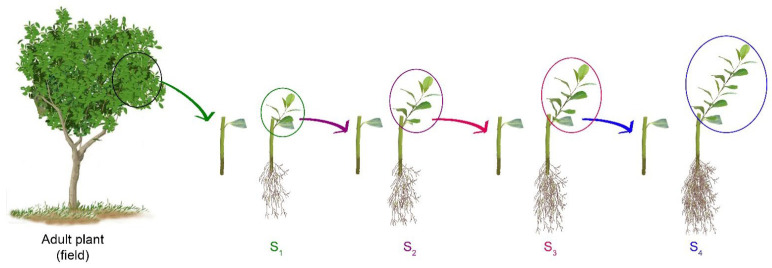
Rejuvenation of *Ilex paraguariensis* adult plants by serial stem-cutting propagation. For the series S_1_ plant production, current-year branches collected from adult plants growing in the field were harvested from the inner canopy at the end of the autumn flush of growth following the protocol developed by Tarrago et al. [7]. For the series S_2_ to S_4_, branches were collected from the series S_1_ to S_3_ propagated plants grown under greenhouse conditions.

Figure 1 illustrates the establishment of a rejuvenated population of *Ilex paraguariensis* using a serial stem-cutting propagation method. Our previous findings indicate that leaves are vital for the adventitious root formation during the first thirty days of incubation [7]. Additionally, they play a crucial role in bud sprouting and shoot development in the following sixty days.

**Figure 2:**
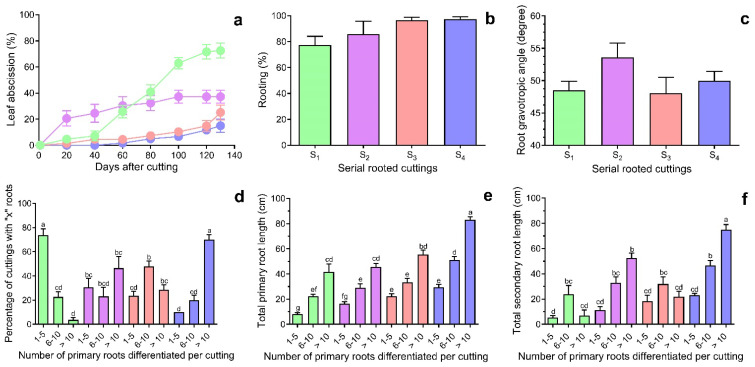
The effect of rejuvenation on adventitious root formation in *Ilex paraguariensis* softwood cuttings after 130 days of IBA (20 mM) and quercetin (500 μM) treatments. (**a**) Leaf abscission; (**b**) Adventitious rooting; (**c**) Primary root gravitropic angle; (**d**) Number of primary roots formed per rooted cutting; (**e**) Total length of primary roots; (**f**) Total length of secondary roots. Bars indicate mean ± SEM (n = 30). According to the Tukey Multiple Comparison Test, different letters indicate significant differences (α = 0.05). Green, purple, pink, and blue bar colours represent S_1_, S_2_, S_3_, and S_4_ serial rooted cuttings, respectively.

Figure 2a shows that rejuvenation affects leaf abscission, which is higher in S_1_ and S_2_ concerning the reinvigorated S_3_ and S_4_ series. After eighty days of incubation, 40.9 ± 5.4% of S_1_ cuttings lost their leaves, significantly different from S_3_ (7.4 ± 1.5%) and S_4_ cuttings (5%) at *p* < 0.01 (Dunnett’s Multiple Comparisons Test). At day 130, the rate of adventitious root formation ranged from 77.5 ± 6.6% for S_1_ cuttings to 97.5 ± 1.7% for S_4_ cuttings (Figure 2b). Although no significant differences in the root gravitropic setpoint angle were detected, a slight increase was noted in the S_2_ and S_4_ series (Figure 2c).

**Figure 3:**
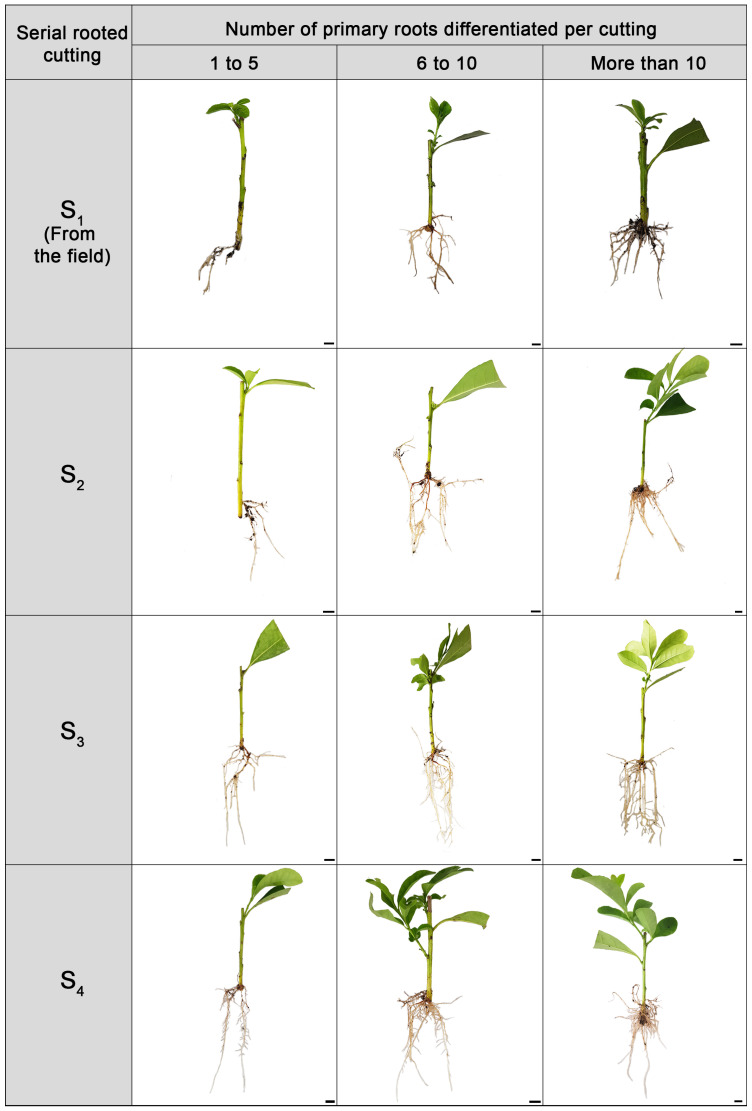
The effect of rejuvenation on *Ilex paraguariensis* root and shoot growth after 130 days of adventitious root induction with IBA (20 mM) and quercetin (500 μM) treatments. In all cases, bars indicate 1 cm.

The cuttings were thoroughly categorised into three groups based on the number of primary adventitious roots formed (Figure 3). This classification allows for a more precise understanding of their developmental patterns. These groups were as follows: those yielding between 1 and 5 roots, those producing 6 to 10 roots, and those generating more than 10 roots (Figure 2d–f). This classification seeks to elucidate the observed patterns of root development and enhance our analysis. Due to rejuvenation, over 70% of S_1_ cuttings developed between 1 and 5 primary roots (Figure 2d). In contrast, 22.6 ± 4.4% produced between 6 and 10 roots, while only 3.7 ± 1.9% yielded more than 10. On the other hand, 90% of S_4_ cuttings during the same period produced between 6 and 10 roots (20 ± 4.1%) and more than ten adventitious roots (70 ± 4.1%) per rooted explant. In all cases, the total length of the primary roots showed a positive correlation with the number of differentiated roots per cutting (Figure 2e). As the number of roots increased, the total length also grew, reaching its maximum in S_4_ cuttings with more than ten roots. The overall length of the secondary roots exhibited a similar growth pattern. (Figure 2f).

**Figure 4:**
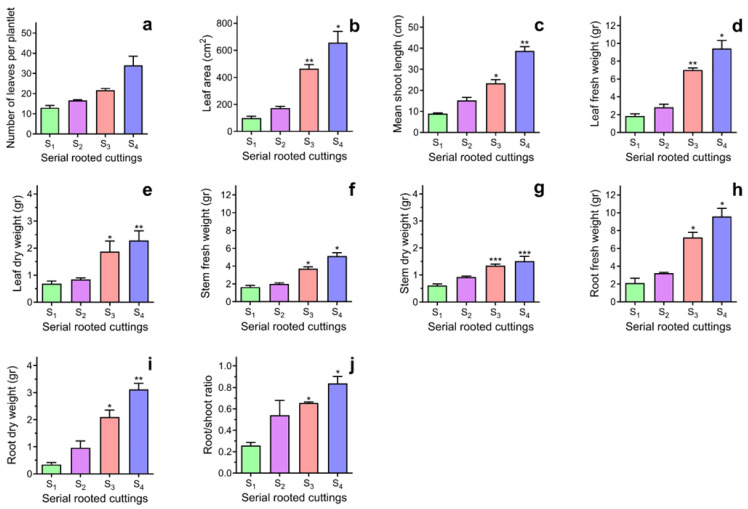
The effect of rejuvenation on the growth of *Ilex paraguariensis* plantlets after 365 days of IBA (20 mM) and quercetin (500 μM) treatments. (**a**) Mean number of leaves differentiated per plant; (**b**) Leaf area; (**c**) Shoot length; (**d**) Leaf fresh weight; (**e**) Leaf dry weight; (**f**) Stem fresh weight; (**g**) Stem dry weight; (**h**) Root fresh weight; (**i**) Root dry weight; (**j**) Root to shoot (leaf + stem) ratio. Bars indicate mean ± SEM (n = 30). According to the Dunnett Multiple Comparison Test, one, two, and three asterisks denote significant differences concerning S_1_ at *p* < 0.05, 0.01, and 0.001. Green, purple, pink, and blue bar colours represent S_1_, S_2_, S_3_, and S_4_ serial stem cuttings.

The number of rooted cuttings with shoots increased from S_1_ (80 ± 6.3%) to S_4_ (92 ± 3.7%). As expected, the increase in primary root numbers and the lengths of primary and secondary roots enhanced the root structure, which expanded its ability to absorb water and nutrients, ultimately promoting plant growth. Figure 4 shows that the S_3_ and S_4_ plants had higher average values for leaf number, leaf area, shoot length, fresh and dry leaf weight, fresh and dry root weight, and root-to-shoot ratio after 365 days from the IBA induction. Except for the average number of leaves developed per plant, the other parameters exhibited significant differences compared to the S_1_ and S_2_ plants. The promotion of growth parameters did not impact the root-to-shoot ratio, and the plants propagated serially displayed more vigorous growth as the ageing reversal process progressed.

### 2.2. The Effects of Rejuvenation on Free-Hormones and Trehalose Content

**Figure 5:**
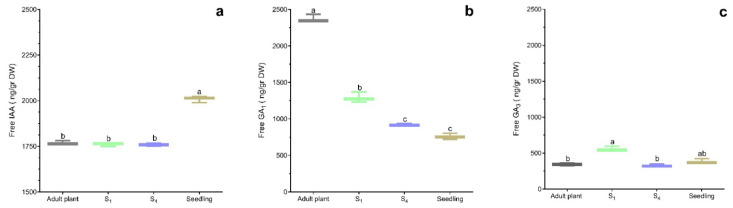
Box plots display the descriptive statistics of indole-3-acetic acid, gibberellin A_1_, and gibberellin A_3_ content in the leaves of adult plants, S_1_, S_4_, and seedlings. Indole-3-acetic acid (**a**), gibberellin A_1_ (**b**), and gibberellin A_3_ (**c**) are presented in their active (free) form.

Before cutting, the endogenous IAA content was significantly lower in the adult, S_1_, and S_4_ leaves (*p* < 0.01) than in the seedlings (Figure 5a). The GA_1_ content decreased from the adult plant to the S_1_ (*p* < 0.01), and S_4_ (*p* < 0.001) serially propagated plants, with the lowest levels found in the leaves of the seedlings (*p* < 0.0001). The GA_3_ content displayed a less distinct pattern, indicating variability in the results. The S_1_ plants and seedlings had higher GA_3_ levels than the adult and S_4_ plants.

The trehalose content varied significantly, ranging from 2210 ± 316 μg/g DW in adult plants to 83 ± 14 μg/g DW in seedlings (*p* < 0.05). Adult plants exhibited a 26-fold increase in trehalose content compared to the juvenile ones. While no statistical differences were observed, S_1_ plants had trehalose levels of 757 ± 97 μg/g DW, compared to S_4_ plants, which had levels of 726 ± 116 μg/g DW. Both levels indicate an approximately threefold decrease compared to adult plants.

### 2.3. Plant Reversal Age Estimation by miRNA Expression

**Figure 6:**
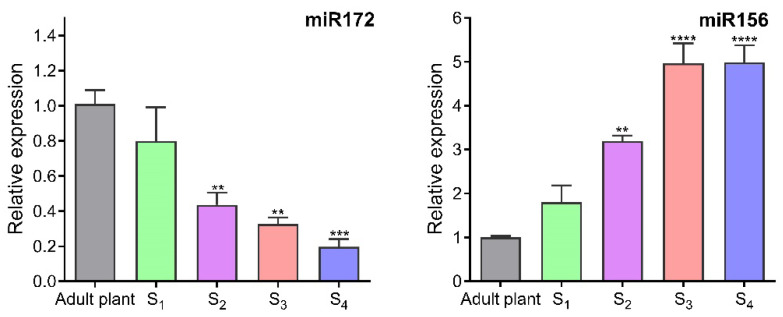
Impact of serial cutting propagation on miR172 and miR156 expression in mature leaves of *Ilex paraguariensis*. The values were normalised using *IpRTF1* as the reference gene. The quantitative real-time polymerase chain reaction (RT-qPCR) results are presented as means ± SEM from three biological replicates. Dunnett’s multiple comparison test indicates statistical significance with two, three, or four asterisks for *p* < 0.01, *p* < 0.001, and *p* < 0.0001.

To evaluate the reversion grade of physiological age in plants propagated through serial cutting, we analysed the expression levels of miR172 and miR156. Figure 6 illustrates that the relative expression of miR172 in mature leaves decreased with the progression of serial propagation. Specifically, the expression levels of miR172 in S2, S3, and S4 plants decreased by factors of 2.3, 3.1, and 5.1, respectively. Conversely, the miR165 expression increased due to rejuvenation, showing statistically significant differences for S2, S3, and S4 plants.

### 2.4. Expression of Genes Involved in Adventitious Root Formation

**Figure 7:**
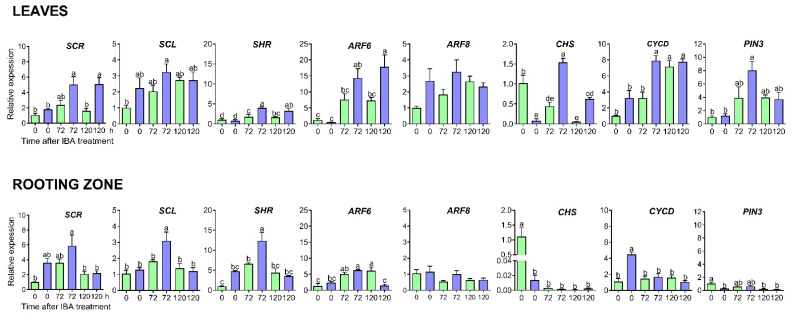
Expression patterns of candidate genes associated with rooting competence in leaves and rooting zone of S_1_ (green) and S_4_ (blue) cuttings. *SCR*, *SCARECROW*; *SCL1*, *SCARECROW-LIKE*; *SHR*, *SHORT ROOT*; *ARF6*, *AUXIN RESPONSE FACTOR 6*; *ARF8*, *AUXIN RESPONSE FACTOR 8*; *CHS*, *CHALCONE SYNTHASE*; *CYCD*, *CYCLIN-DEPENDENT PROTEIN KINASE INHIBITOR SMR6*; *PIN3*, *AUXIN EFFLUX CARRIER COMPONENT 3*. Bars indicate mean ± SEM. Significant differences, determined by the Tukey Multiple Comparison Test at *p* < 0.05, are indicated by lowercase letters. The *IpRTF1* gene was utilised as an internal control.

Figure 7 illustrates the expression levels of selected genes in the leaves and stem bases of S_1_ and S_4_ cuttings, measured immediately after IBA treatment and again at 72 and 120 h later. The expression patterns of GRAS transcription factor-related genes, specifically *SCARECROW (SCR*), *SCARECROW-LIKE* (*SCL1*), and SHORT ROOT (*SHR*), showed an increase in the leaves, particularly pronounced in the S_4_ cuttings. A significant peak in expression was observed at the stem base of S_4_ cuttings 72 h after induction. Additionally, the accumulation of *SCL* and *SHR* transcripts was statistically different compared to S_1_ cuttings.

The *AUXIN RESPONSE FACTOR* 6 (*ARF6*) transcript accumulation was notably higher in the leaves, increasing 2.4-fold in S_4_ compared to S_1_ (*p* < 0.05) at 120 h after induction. However, in the rooting zone of S_4_ cuttings, *ARF6* expression decreased by more than four-fold concerning S_1_ (*p* < 0.01). The levels of *ARF8* transcripts did not show significant variation across different organs or cutting series.

The *CHALCONE SYNTHASE* (*CHS*) expression increased in the leaves of rejuvenated S_4_ cuttings after 72 h. The transcript variation pattern was similar in the rooting zone of both cutting series, peaking immediately after auxin treatment but declining significantly. In the leaves, the expression of *CYCLIN-DEPENDENT PROTEIN KINASE INHIBITOR SMR6* (*CYCD*) and *AUXIN EFFLUX CARRIER COMPONENT 3* (*PIN3*) was most prominent at 72 and 120 h. In the rooting zone, the highest accumulation of *CYCD* transcripts occurred immediately after auxin treatment in S_4_ cuttings, while *PIN3* expression was highest in S_1_ cuttings.

### 2.5. Small RNA Sequencing and miRNA Identification

Twelve libraries were constructed, corresponding to the four physiological ages, each created in triplicate. A total of 620,054,754 raw reads were produced, and all cases achieved a Q30 quality score greater than 94.9%. Low-quality reads and poly-A sequences were removed, each library retaining between 26,936,657 and 41,340,614 clean reads. More than 91% of the *I. paraguariensis* genome was mapped using these cleaned reads (Table S1). These results validate the overall quality of the reads.

Subsequent analysis identified 2099 known mature miRNAs grouped into 96 families (Table S2) and predicted 170 novel mature miRNAs, designated in this work as ipa-miR-n ‘number’, such as ipa-miR-n85 (Table S3).

### 2.6. Changes in miRNAs Expression Among Plants at Different Physiological Ages, Target Prediction and Functional Analysis

A differential expression analysis was conducted using the Wald test, implemented in the DESeq2 package in R. The analysis compared the expression levels of miRNAs in adult plants with those in S_1_, S_4_, and seedlings. The relative expression of 10 selected miRNAs between adult plants and S_4_ was measured using RT-qPCR as the reference method to validate these results. The correlation coefficient (R^2^) was 0.95, confirming the reliability of the findings (Figure S1). The differential expression analysis was conducted twice: first using the counts of the 96 known miRNA families (Table S4) and then the counts of the 170 novel miRNAs (Table S5). A differentially expressed miRNA (DE-miRNA) was defined as one with a Fold Change ≥ 2 or ≤0.5 (equivalent to Log2 Fold Change ≥ 1 or ≤−1) and a *p*-value < 0.05 (Tables S6 and S7).

In comparing adult plants and S_1_, we identified 37 differentially expressed miRNAs (DE-miRNAs), with 21 showing upregulation and 16 downregulation. When comparing adult plants to S_4_, the number of DE-miRNAs increased to 49, of which 25 were overexpressed and 24 were repressed. As anticipated, the comparison between adult plants and seedlings revealed the most significant differences, with 80 miRNAs displaying differential expression—44 were increased, while 36 were suppressed. This analysis included both known miRNA families and novel predicted miRNAs. For detailed information, refer to Table S8.

**Figure 8:**
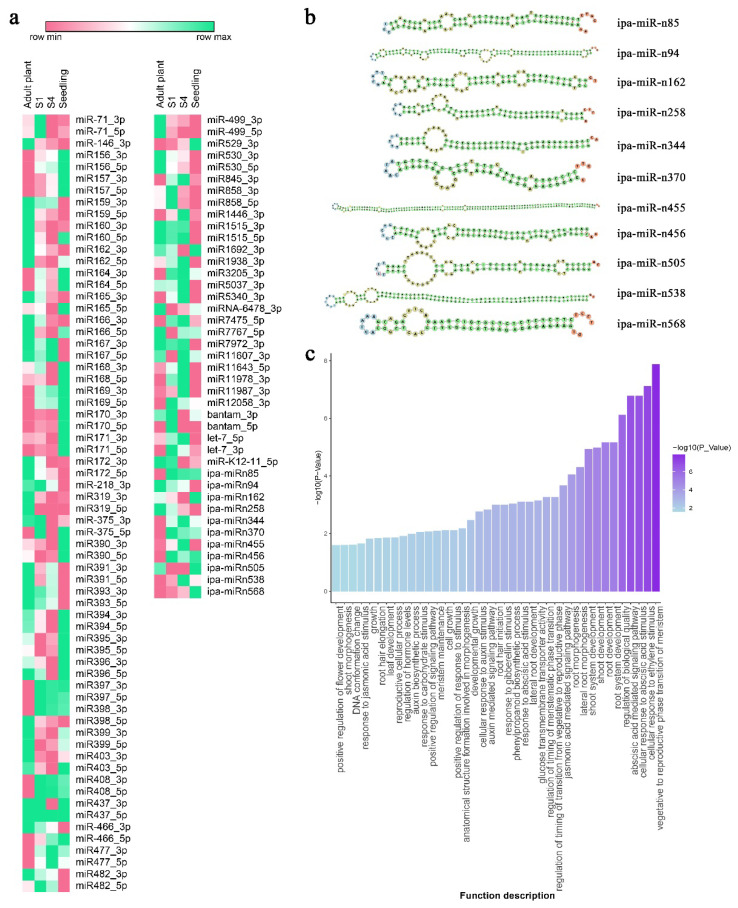
Computational analysis of differentially expressed miRNAs in *Ilex paraguariensis* leaves. (**a**) Differential expression analysis of 96 known miRNA families and 11 novel predicted miRNAs; (**b**) Secondary structure of novel miRNAs precursors; (**c**) Functional enrichment analysis of gene target of all DE-miRNAs (adult vs. S_4_ plants) utilising gene ontology (GO). The significance levels of GO terms were determined using the hypergeometric test, with *p*-values represented as −Log10 when comparing the adult plant to S_4_.

Similar expression changes can be observed in some miRNAs by simultaneously analysing the changes across all four developmental stages. Below are examples illustrating these patterns without being exhaustive. In the physiological rejuvenation sequence, the field plant represents the most mature stage, followed by the two vegetative propagation reversal series, S_1_ and S_4_, while the seedling is the most juvenile stage. The miRNAs 156, 157, and 169 continuously increase in expression from adult plants through S_1_ and S_4_, ultimately reaching the seedling stage. In contrast, the known miRNAs 172, 499, and 530, along with the predicted ipa-miR-n94, show a consistent decline in expression levels from adult plants to S_1_, S_4_, and seedlings. The expression levels of miRNAs 397, 398, and 408, along with the novel ipa-miR-n85, do not show statistically significant differences among the S_1_, S_4_, and seedling stages. However, these levels are notably higher than those observed in the adult plant. Conversely, miR319 behaves differently; it remains stable during the S_1_, S_4_, and seedling stages but shows a significant decrease compared to the adult plant (Figure 8a).

This study examines the rejuvenation of *I. paraguariensis* resulting from serial vegetative propagation. Our approach involves a comparative analysis between mature plants and those that have reached the S_4_ stage, representing the most advanced rejuvenation level achieved through this propagation method. Then, the potential functions of DE-miRNAs between the adult and S4 plants were assessed by predicting their target genes, which led to the identification of 573 genes. Among these, 420 genes were targeted by 38 DE-miRNA families, and 153 were targeted by 11 differentially expressed novel DE-miRNAs (Figure 8b; Table S9). Subsequently, a Gene Ontology (GO) enrichment analysis was conducted to explore the biological functions associated with this set of 573 target genes. The complete GO biological network, which includes categories for molecular function, biological process, and cellular component, is presented in Figure S2 and Table S10. The GO terms related to Biological Processes were analysed to connect the earlier biometric results with the underlying molecular processes and changes in miRNA expression profiles. This analysis revealed that the most relevant GO terms included the transition from the vegetative to the reproductive phase of the meristem, cellular responses to hormone stimulus, regulation of biological quality, root system development, root development, and shoot development, among others (Figure 8c; Table S11).

## 3. Discussion

The strong recalcitrance of *I. paraguariensis* adult plants limits the vegetative propagation of selected genotypes that exhibit desirable traits, such as stress resilience and specific metabolite composition. The transition from juvenile to adult stages typically involves a gradual decline in rooting capacity and root structure, impacting overall plant growth and reducing survival rates in field conditions. The timing of transitions between these stages, known as phase transitions, varies significantly depending on the species [25]. In annual species such as *Arabidopsis thaliana* and *Zea mays*, the microRNAs miR156 and miR172 play significant roles in regulating phase transitions [26,27]. These miRNAs are key components of the ageing pathway and work in succession to regulate the onset of reproductive competency [28]. In these species, the expression of miR156 is very high during the seedling stage but gradually decreases as the plant transitions from juvenile to adult stages. Conversely, miR172 exhibits the opposite expression pattern. A similar miRNA expression relationship has also been observed in perennial woody species with well-defined juvenile and adult phases, such as *Acacia confusa*, *Acacia colei*, *Eucalyptus globulus*, *Hedera helix*, and *Quercus acutissima* [29]. MicroRNA 156 is widely distributed across nearly all major plant taxa, and its function in regulating vegetative phase change is conserved throughout the entire plant kingdom [25]. We measured the expression levels of both microRNAs in the leaves of *I. paraguariensis* and observed variations during the serial propagation process. Our results showed that the expression of miR156 increased while miR172 decreased throughout this sequence. We found an inverse correlation between the levels of miR156 and miR172 in adult and rejuvenated tissues. This variation confirmed that the transition of adult *I. paraguariensis* plants back to a juvenile phase occurs after serial rooted cuttings, enabling the plants to regain juvenile characteristics.

Plant hormones primarily influence the vegetative phase change by interacting with cytokinins and gibberellins [30]. This hormonal signalling pathway is believed to be similar in annual and perennial species [31]. Furthermore, the interactions between plant hormone networks and the miR156/SPL module are more noticeable at the protein level, where SPL proteins physically interact with components of brassinosteroid, strigolactone, abscisic acid, and cytokinin signalling. Additionally, SPL transcription factor and cytokinin signalling work together to promote the miR172 expression [30,32]. The observation that hormones function downstream of miR156 and miR157, coupled with the absence of evidence linking variations in hormone levels to vegetative phase change, suggests that plant hormones are primarily involved in the expression of vegetative phase change rather than its timing [32]. Our findings suggest that lower GA_1_ levels may be associated with age reversion, although further studies are needed to address existing gaps. The reduction in GA_1_ concentration appears to be associated with the loss of flowering in rejuvenated S_4_ plants. Furthermore, the inconsistent effects of GA_3_ may relate to our previous findings from both in vitro and in situ experiments, which indicate that gibberellins with different structures can antagonistically affect the vegetative growth of *I. paraguariensis* [33,34]. Specifically, 1,2-didehydro-gibberellins, such as GA_3_ and GA_7_, inhibit shoot development, whereas their counterparts, which lack a double bond at ring A (GA_1_ and GA_4_), promote vegetative growth.

Sugars, mainly sucrose, glucose, and fructose, may promote the transition to the reproductive phase (the flowering time) by repressing the A and C genes associated with MIR156 [16]. These genes produce mature miR156, suggesting that carbohydrate levels may influence age-dependent pathway output [35]. Then, as the plant matures and accumulates more sugars, the expression of miR156 is repressed, which allows the vegetative phase transition to begin [36]. More recently, Ponnu and colleagues [37] reported that the trehalose 6-phosphate (T6P) pathway also affects vegetative phase change in *Arabidopsis thaliana* by suppressing miR156 expression, which, in turn, modulates the levels of its target transcripts, the *SPL* genes. Likewise, T6P is crucial for regulating embryonic maturation, axillary bud growth, and leaf starch degradation [38]. Trehalose 6-phosphate, which is produced during trehalose biosynthesis, serves as a signalling metabolite that conveys the sucrose status to downstream signalling pathways. In plants, trehalose is produced from glucose 6-phosphate and UDP-glucose, using T6P as an intermediate. The enzyme TREHALOSE PHOSPHATE SYNTHASE 1 catalyses the formation of T6P, which is then dephosphorylated into trehalose by TREHALOSE PHOSPHATE PHOSPHATASES [39]. It is still unclear whether the actions of T6P are related to trehalose and whether endogenous trehalose may function as a signalling molecule in conjunction with T6P to regulate plant growth, development, and stress responses [39]. Our findings show that the trehalose content in the leaves of adult *I. paraguariensis* plants is 26 times higher than that found in seedlings. We also observed that trehalose levels in rejuvenated plants decrease by about threefold. This notable variation may be associated with miR156 and miR172 expression changes, indicating its possible role in reverting to a more juvenile state.

The analysis of miRNAs transcriptomic profiles between adult and S_4_ plants, conducted via RNA sequencing, reaffirmed the predicted expression patterns of miR156 and miR172. Furthermore, the functional enrichment analysis identified several differentially expressed miRNAs that influence the timing of the transition from the vegetative to the reproductive phase. Notably, miR169 and miR399 are linked to the flowering time control, while miR157, miR394, miR529, and miR530 are associated with the meristem transition. Furthermore, miR1446, which plays a role in reproductive cellular processes, was also found to be differentially expressed. The miR169 family is one of the most abundant miRNA families in *Arabidopsis* and plays a key role in regulating flowering time in response to various stresses by targeting the Nuclear Factor Y, Subunit A (NF-YA) transcription factor gene family [40]. The miR169/NF-YA module functions independently of the miR156/miR172 pathway and is essential for promoting early flowering under stressful conditions [41]. Additionally, miR394 and miR399 control flowering time in response to abiotic stress [42,43].

The microRNAs miR157 and miR529 are closely related to miR156, acting to repress the expression of SQUAMOSA PROMOTER BINDING PROTEINS (SBP/SPL), which control the transition of plants from vegetative to reproductive growth [16,44,45]. Moreover, the expression of miR530 is influenced by circadian rhythms, suggesting its importance in the context of phase change [46]. Lastly, miR1446 has been linked to vernalisation in the dormant buds of *Prunus persica* [47].

Our research findings also reveal a significant overexpression of miR398 and miR408. In conjunction with miR156, these microRNAs are closely associated with the sugar signalling pathway and phase change transitions [48,49]. This observation underscores their potential importance in advancing our research objectives.

Finally, we analysed the expression of several genes associated with the early stages of adventitious root induction in the leaves and stem bases of S_1_ and S_4_ cuttings following treatment with IBA. Our results indicate that the expression levels of GRAS transcription factor-related genes—specifically *SCR*, *SCL*, and *SHR*—were significantly elevated in the leaves and stem bases of the rejuvenated S_4_ cuttings 72 h after induction. This finding highlights the spatial and temporal occurrence of early molecular events associated with forming a new root meristem before cell division [50]. Notably, the accumulation of *SCL* and *SHR* transcripts in the stem bases of the S_4_ cuttings was significantly greater than that observed in the S_1_. Additionally, we detected a significant accumulation of ARF6 transcripts in the S_4_ leaves, which play a critical role in auxin signalling in correspondence with ARF8 and ARF 17 [51]. These transcriptomic changes may be linked to the promoter effect of the rejuvenation process, which contributes to the enhancement of adventitious root proliferation in *Ilex paraguariensis* cuttings. As a result, increasing the number and length of both primary and secondary roots improved the overall root structure and promoted plant growth. The rejuvenated S_3_ and S_4_ plants showed higher average values in leaf number, leaf area, shoot length, fresh and dry leaf weight, fresh and dry root weight, and root-to-shoot ratio than the S_1_ and S_2_ plants. While the growth parameters were enhanced, this did not affect the root-to-shoot ratio. This feature is essential for achieving a balance in growth and plays a significant role in plant survival during the establishment phase of the plantation in the field. Additionally, the plants propagated serially exhibited more vigorous growth as the ageing reversal process advanced.

## 4. Materials and Methods

### 4.1. Establishing a Plant Population with Different Physiological Ages

A thirty-year-old *Ilex paraguariensis* cultivar was used as the donor mother plant. Current-year branches collected from plants growing in the field were used to initiate a serial cutting propagation (Figure 1). For the series S_1_ plant production, branches (35–40 cm long) were harvested from the central structure of each plant (inner canopy) at the end of the autumn flush of growth following the protocol developed by Tarrago et al. [7]. For the series S_2_ to S_4_, branches were collected from the S_1_–S_3_ propagated plants grown under greenhouse conditions. Softwood cuttings (10–12 cm long, 3–5 mm diameter) consisted of six to nine nodes in which the uppermost mature leaf was cut in half and retained while the lower six to eight leaves were removed. For rooting, the basal part of the cuttings was immersed for 4 min in IBA 20 mM (Sigma-Aldrich^®^, St Louis, MO, USA) ethanolic solution (50% *v*/*v*), followed by a 60 min treatment with quercetin 500 µM (Sigma-Aldrich^®^) according to Tarragó et al. [8]. Finally, cuttings were dipped in a talc mixture containing 10% sucrose and 10% Captan fungicide before being set into 150 cc trays containing composted pine bark plus 0.5 g of controlled-release micro-fertiliser (Plantacote^®^, N-P-K-Mg, 14-8-12.5-1.2), and grown for 130 days in a growth chamber with a day/night air temperature of 25–27/20–22 °C and a substrate temperature of 22–25 °C, provided by a hotbox. Relative humidity was maintained at 90% during the first 7 days by a misting device and then decreased gradually until 65%.

Afterwards, to evaluate the rejuvenation process, the miR156 and miR172 expression in mature leaves of adult field plants, serial rooted cuttings (S_1_, S_2_, S_3_, and S_4_), and seedlings were assessed using reverse transcription followed by quantitative real-time polymerase chain reaction (qRT-PCR).

### 4.2. Measurements for Growth Traits

The rooting rate, number of roots per cutting, root architecture, and root gravitropic setpoint angles were measured after 130 days of incubation. Additionally, leaf abscission was monitored every ten days to assess its impact on root formation and bud sprouting in *I. paraguariensis* softwood cuttings, where leaf retention is critical. The root gravitropic setpoint angle was determined using ImageJ2 software v2.0.0 following the procedure described by Rueden et al. [52]. Finally, cuttings were sorted into three categories in each propagation series based on the number of adventitious primary roots formed: 1–5, 6–10, and more than 10 primary roots.

In addition, measures were taken for bud sprouting, shoot length, number of phytomers, number of shoots, leaf area, and biomass partitioning in one-year-old propagated plants, with three plants analysed per rejuvenation series.

### 4.3. Plant Reversal Age Estimation by miRNAs RT-qPCR

Mature leaves from adult field plants and serial rooted cuttings (S_1_, S_2_, S_3_, and S_4_) were harvested and immediately frozen in liquid nitrogen until processing. Then, total RNA was extracted using the mirVana^TM^ miRNA Isolation kit (ThermoFisher Scientific Inc., Waltham, MA, USA) following the manufacturer’s instructions. Stem-loop primers (Table S1) were used to synthesise cDNA from the miR156a-5p (available at https://www.mirbase.org/mature/MIMAT0000166, accessed on 24 February 2025) and miR172a (available at https://www.mirbase.org/mature/MIMAT0000203, accessed on 24 February 2025). The RNA polymerase-associated protein rtf1 (RTF) transcript (GenBank: KU886201) was employed as an internal control [53]. Briefly, for each miRNA, 0.5 μL of 1 μM of the specific stem-loop primer was mixed with 0.5 μg total RNA in individual reactions, and were added 2 μL ImProm-II™ 5X Reaction Buffer (Promega Corp., Madison, WI, USA), 1.2 μL MgCl2 (25 mM), 0.5 μL dNTP Mix (10 mM), and 0.5 μL ImProm-II™ Reverse Transcriptase (Promega Corp., Madison, WI, USA). The reaction mixtures were adjusted to a final volume of 15 μL. Oligo dT_20_ was used as primers in the reverse transcription of RTF mRNA. The process started with an initial step at 16 °C for 30 s, followed by 60 cycles at 30 °C for 30 s, 42 °C for 30 s, and 50 °C for 1 s. Finally, an enzymatic inactivation phase at 85 °C for 5 min was completed [54]. The resultant reaction solution was then stored at −20 °C until use.

Each quantitative real-time PCR (qPCR) reaction was assembled with 80 ng cDNA, 7.5 μL of 2X SYBR^®^ Select Master Mix (Applied Biosystems, Waltham, MA, USA), and 300 nM of the corresponding primer pair (Table S1) in a final volume of 15 μL. All reactions were performed in technical triplicate using a Line Gene 9600 Real-Time PCR System (Peet Lab, Hangzhou, China) with an initial step at 95 °C for 10 min, 40 cycles of 95 °C for 15 s, and 60 °C for 1 min. The results were processed using the ΔΔCT method to calculate the expression’s relative quantitation (RQ) [55]. Previously, an assay was conducted to standardise the sampling process by determining miRNA expression in leaves at different maturation stages in the same plant and growing flash. The leaves included in the assay were 1, 6, 12, and 15, with leaf one being the furthest from the shoot apices. The results showed that leaf 1 had the highest and most stable expression among the tested leaves; hence, it was selected for the age estimation and sequencing experiments.

### 4.4. RNA Isolation, Library Construction, and Sequencing

Total RNA was extracted from adult field plants, serial rooted cuttings (S_1_, S_4_), and seedlings mature leaves as described in 4.3. RNA integrity was performed using the Agilent 2100 Bioanalyzer system with an RNA nano/pico chip platform (Agilent Technologies, Santa Clara, CA, USA). Only samples with a RIN value ≥ 7 (RNA Integrity Number) were used in the following procedures. Purity was assessed by the A260/A280 and A260/A230 absorption ratios with a NanoDrop™ 2000 spectrophotometer (Thermo Fisher Scientific, Waltham, MA, USA). Concentrations were determined using Qubit™ RNA BR Assay Kit (Thermo Fisher Scientific). An ARN size selection in the 13–40 nt range was performed using the BluePippin method. The NEBNext Small RNA Library Prep Set for Illumina was used to construct 12 libraries sequenced on the Illumina Novaseq 6000 platform to produce 2 × 51 nucleotides paired-end reads (Macrogen, Seoul, Korea).

### 4.5. RNA-Seq Data Processing

The quality of the raw reads was assessed using FastQC. Then, we continue with the pipeline proposed by Garg and Varshney [56], with some modifications described below. Low-quality reads and adaptor sequences were removed by Trimmomatic v0.39 with the “PE” module, and parameters ILLUMINACLIP:NEBNext_small_RNA.fasta:2:30:10 and SLIDINGWINDOW:4:15. Poly-A reads were trimmed and shorter than 18 nt, and longer than 34 nt were discarded using Cutadapt v2.8, setting -a “A{20}” -m 18 -M 34. Filtered reads obtained from each sample were first combined into a single FASTA file and then mapped on the *I. paraguariensis* genome [57] by the Bowtie v1.2.3 software to determine whether the sequenced RNA molecules originate from this plant or may proceed from contamination.

In order to obtain the known miRNAs, all combined reads were collapsed into unique tags using the FASTX-Toolkit 0.0.14. Thus, the task is simplified by identifying each miRNA through a single sequence that represents all identical reads. Then, these unique tags were mapped on the I. paraguariensis ribosomal (rRNAs) and transfer (tRNAs) RNAs (available at https://www.ncbi.nlm.nih.gov/datasets/gene/GCA_963454935.2/ (accessed on 24 February 2025), and also on plant sequences of small nuclear RNAs (snRNAs) and small nucleolar RNAs (snoRNAs) (available at https://rfam.org/, accessed on 24 February 2025). The unique tags aligned with these sequences were eliminated, while the unaligned reads were saved for subsequent mapping to the mature miRbase database v22.1, which contains published miRNA sequences and their annotations. This step identifies the aligned reads as members of known miRNA families.

On the other hand, the unique tags not aligned against the miRbase were employed for novel miRNA prediction by the miRDeep-P package (v1.3). In the first step, these sequences were aligned to the *I. paraguariensis* reference genome using Bowtie with 0 or 1 mismatch. The SAM files were converted to blast format, and subsequently, only those alignments that meet the criteria of 100% sequence identity, full-length alignment, and no more than 15 matches were retained. Among these, the reads mapped on annotated features (Exons, CDS, etc.) already recognised for this plant were dismissed. The surviving reads were then used to extract potential precursor sequences from the reference genome, with a 250 bp window size. These excised sequences were analysed with the RNAfold utility from the Vienna RNA package to predict their secondary structures. Potential precursor sequences were used to align the previously filtered reads and generate miRNA signatures. In the final step, miRDeep-P combines this information, predicts novel miRNAs, and removes predicted miRNAs that do not meet the criteria of plant miRNAs.

The potential functions of differentially expressed miRNAs were investigated through their target genes. For this reason, the online portal psRNATarget (release 2017) was used to find the miRNA targets with a cutoff value of E ≤ 3.0. The BiNGO tool (v3.0.5) was utilised to perform the GO enrichment test in Cytoscape v3.10.3, employing the hypergeometric test. The Benjamin Hochberg method was used to adjust raw *p*-values for multiple tests. A high-resolution GO map can be found in Supplementary Figure S2. The RNA secondary structure was calculated and graphed using the ViennaRNA Web Services (available at http://rna.tbi.univie.ac.at/forna/, accessed on 24 February 2025).

### 4.6. Differential Expression Analysis of miRNAs

Using Bowtie v1.2.3, filtered and cleaned reads of each sample were mapped on the known and novel miRNAs found in this work to obtain the counts for each miRNA in every sample. The known miRNAs were grouped into families for further study. Next, a differential expression analysis was conducted using DESeq2, contrasting the adult plant vs. S_1_, S_4_ or seedling. First, miRNA families with low counts (the total counts across all samples being less than 100) were discarded. The remaining counts were normalised using the median of ratios method. Differentially expressed miRNAs were identified through the Wald test, applying filters for a Fold Change of ≥2 or ≤0.5 (Log2 Fold Change of ≥1 or ≤−1) and a *p*-value of <0.05, except for the miRNAs included in the small RNA sequencing validation. Heat maps were created using the Morpheus web server (available at https://software.broadinstitute.org/morpheus/, accessed on 1 April 2025) based on the counts for each sample.

In order to validate the DEmiRNAs calculated by these methods, 10 miRNA families with different expression profiles between adult and S_4_ plants were selected (repressed, overexpressed, and without changes). RT-qPCR was the reference method. Individual miRNA expression was quantified in triplicate as described in Section 4.3, using the primers detailed in Supplementary Table S12.

### 4.7. Expression of Genes Involved in Adventitious Root Formation

Eight genes associated with the formation of adventitious roots, including SCARECROW (SCR), SCARECROW-LIKE 3 (SCL3), SHORT-ROOT (SHR), AUXIN RESPONSE FACTOR 6 (ARF6), AUXIN RESPONSE FACTOR 8 (ARF8), CHALCONE SYNTHASE (CHS), CYCLIN-DEPENDENT PROTEIN KINASE INHIBITOR SMR6 (CYCD), and AUXIN EFFLUX CARRIER COMPONENT 3 (PIN3) were identified from the Ilex paraguariensis transcriptome [58] (Supplementary Table S13). After treatment with IBA, transcript levels in the leaves and basal stems (rooting zone) were analysed using RT-qPCR immediately after the treatment and at 72 and 120 h of the induction stage.

Primers were designed using the Primer3Plus software v3.3.0 (https://www.primer3plus.com/). Individual gene expressions were quantified in biological triplicate using the same reaction conditions described in Section 4.3, but cDNA synthesis employed oligo dT20 as primers.

### 4.8. Hormones and Trehalose Determination

Before cuttings, mature leaf samples were collected from adult field plants, serial rooted cuttings (S_1_, S_4_), and seedlings. The samples were immediately frozen in liquid nitrogen and then freeze-dried. Hormones and trehalose quantification were assessed at the Agricultural Biotechnological Research Institute of the National Research Council of Argentina. Analysis of indole-3-acetic acid (IAA), gibberellin A_1_ (GA_1_), and gibberellin A_3_ (GA_3_) was conducted following a modified procedure described by Iparraguirre et al. [59]. Briefly, analysis was performed using liquid chromatography-tandem mass spectrometry (LC-MS/MS) with a Waters ACQUITY UPLC system (Waters, Milford, MA, USA) linked to a Micromass Quattro Ultima™ double quadrupole mass spectrometer (Micromass, Manchester, UK). Data were acquired and analysed using MassLynx™ 4.1 and QuanLynx™ 4.1 software (Micromass, Manchester, UK). For quantification, the values were obtained from a calibration curve previously established with known amounts of each hormone, utilising their pure standards (Sigma, St. Louis, MO, USA) and the ratios of deuterated internal standards. LC–MS/MS was used to detect and quantify trehalose, with maltose as an internal standard, following the method outlined in Kretschmer et al. [60].

### 4.9. Statistical

For each propagation series, 30 cuttings were included, and the experiment was repeated thrice. The results presented are the means ± SEM of the biological replicates. All variables were subjected to analysis of variance (ANOVA) using the statistical programme Prism vs. 10.3.1 (GraphPad^®^, San Diego, CA, USA) software. Three biological replicates were used per treatment for transcriptomic, hormones and trehalose analyses. In RT-qPCR, each sample was run in technical triplicate.

## 5. Conclusions

Our research indicates that the rejuvenation of adult *Ilex paraguariensis* plants, known for their propagation challenges, can be effectively achieved by applying serial rooting of softwood cuttings. The transition from the juvenile to the adult phase in this species is controlled by a gradual increase in the microRNA miR172, which occurs at the expense of miR156. By reversing this ratio, the serial rooting technique not only stimulates the adventitious root formation but also enhances root structure, thereby facilitating the overall growth of the resulting plant and contributing to greater vigour.

These results offer valuable insights that may be applicable to other challenging woody species, including *Eucalyptus globulus*, *Eucalyptus nitens*, *Juglans* spp., and *Hevea brasiliensis*, among others. Implementing a straightforward methodology involving the serial rooting of cuttings has the potential to establish a robust stock of rejuvenated mother plants, which will provide physiologically competent explants. This approach is expected to enhance the efficiency of mass propagation and significantly improve the root architecture and vigour of the resulting plants, thereby increasing their survival rates during the establishment phase of commercial forest.

## Data Availability

We stored the raw sequence in The National Center for Biotechnology Information (NCBI), and they are identified with the BioProject accession number PRJNA1232600.

## Supplementary Materials

The following supporting information can be downloaded at: https://www.mdpi.com/article/10.3390/plants14111668/s1, Figure S1: miRNAs expression correlation analysis; Figure S2: Biological network of gene ontology (GO) terms significantly overrepresented in the DE-miRNA target genes; Table S1: Information about small RNA sequencing; Table S2: Known miRNA families identified. Table S3: Novels miRNA predicted in *Ilex paraguariensis* leaves; Table S4: Counts of known miRNA families; Table S5: Counts of novel predicted miRNAs; Table S6: Differential expression analysis of known miRNA families calculated by DESeq2; Table S7: Differential expression analysis of novel predicted miRNAs calculated by DESeq2; Table S8: Summary of differentially expressed miRNAs; Table S9: DE-miRNAs target genes predicted by psRNATarget (adult plant vs. S_4_); Table S10: BiNGO overrepresentation of Gene Ontology categories of target genes (DE-miRNAs adult plant vs. S_4_); Table S11: Selected Gene Ontology categories related to biometrics results in this paper; Table S12: Primer sequences; Table S13: Primer sequences of selected genes involved in adventitious root formation.
