# Supplementary figures and images for: Rejuvenation of Mature Ilex paraguariensis Plants Through Serial Rooted Cuttings: Exploring the Roles of miRNAs in Reversing Adult Phase, Promoting Root Formation, and Determining Root Structure
Source: Plants (Basel). 2025 May 30;14(11):1668. doi: 10.3390/plants14111668 (PMC12157841; doi:10.3390/plants14111668)

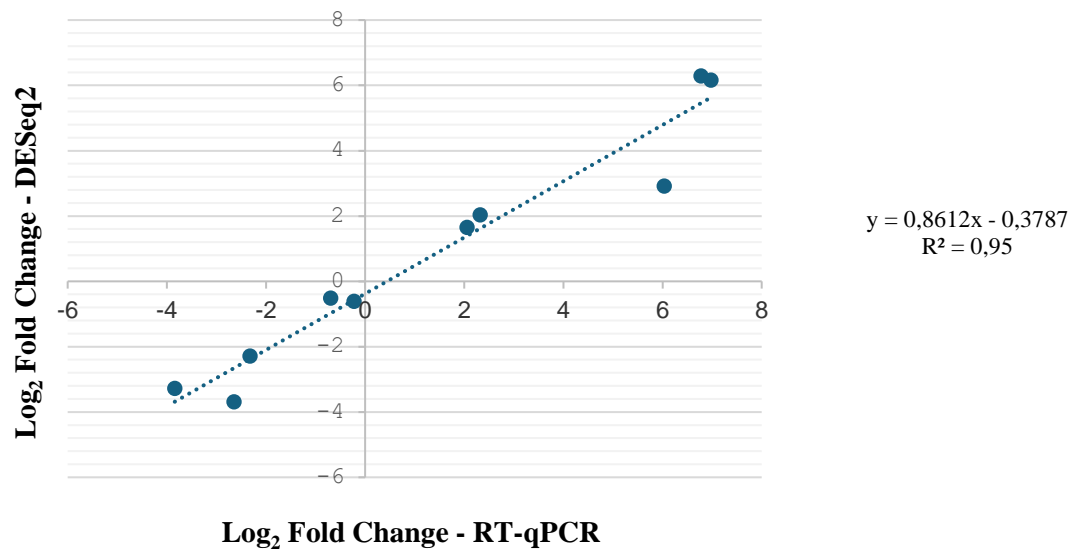

**Figure S1.** miRNAs expression correlation analysis.

Supplement: Supplementary file 1 [file plants-14-01668-s001.zip › Figure S1.pdf]
